# Comparative Analysis of Morphology, Resource Allocation, and Nutritional Characteristics in Populations of *Festuca dolichophylla* Cultivated in the Andean Region of Peru

**DOI:** 10.3390/plants15030474

**Published:** 2026-02-03

**Authors:** Ysai Paucar, Samuel Porfirio Paucar, Flor Lidomira Mejía, Héctor Vladimir Vásquez, Luis Homero Zagaceta, José Américo Saucedo-Uriarte, Ives Yoplac, Enrique Ricardo Flores, José Luis Contreras, Gregorio Fructuoso Argote, Teodoro Bill Yalli, Lucrecia Aguirre

**Affiliations:** 1 Laboratorio de Nutrición Animal y Bromatología de Alimentos, Instituto de Investigación en Ganadería y Biotecnología, Facultad de Ingeniería Zootecnista, Biotecnología, Agronegocios y Ciencia de Datos, Universidad Nacional Toribio Rodríguez de Mendoza de Amazonas, Chachapoyas 01001, Peru; ives.yoplac@untrm.edu.pe; 2 Programa de Estudios de Producción Agropecuaria, Instituto de Educación Superior Tecnológico Público Aurahuá, Castrovirreyna 09735, Peru; spaucar@iestpaurahuahvca.edu.pe; 3 Instituto de Investigación en Ganadería y Biotecnología, Facultad de Ingeniería Zootecnista, Biotecnología, Agronegocios y Ciencia de Datos, Universidad Nacional Toribio Rodríguez de Mendoza de Amazonas, Chachapoyas 01001, Peru; flor.mejia@untrm.edu.pe (F.L.M.); hvasquez@untrm.edu.pe (H.V.V.); luis.zagaceta@untrm.edu.pe (L.H.Z.); jose.saucedo@untrm.edu.pe (J.A.S.-U.); 4 Laboratorio de Ecología y Utilización de Pastizales, Facultad de Zootecnia, Universidad Nacional Agraria La Molina, Lima 15024, Peru; efm@lamolina.edu.pe (E.R.F.); laterra@unalm.edu.pe (L.A.); 5 Escuela Profesional de Zootecnia, Facultad de Ciencias de Ingeniería, Universidad Nacional de Huancavelica, Huancavelica 09001, Peru; jose.contreras@unh.edu.pe; 6 Instituto Nacional de Innovación Agraria, Estación Experimental Illpa, Carretera Puno—Juliaca km 22, Puno 21110, Peru; argoteqgf@gmail.com; 7 Instituto Nacional de Innovación Agraria, Sede Central. Av. La Molina 1981, Lima 15024, Peru; byalli022021@gmail.com

**Keywords:** *Festuca dolichophylla*, grassland, nutritional characteristics, productivity, Peruvian Andes

## Abstract

Grasslands are ecosystems of global importance; in Peru, they represent more than half of the country’s territory. However, few studies have been conducted on high Andean grasslands. The objective was to study morphological, productive, resource allocation, and nutritional characteristics in five populations of *Festuca dolichophylla* grown under similar conditions. Populations that originated from Huancavelica Community and University, Junín, Pasco, and Puno were grown in Huancavelica Community in a randomized block design. After twelve months, a uniformization cut was performed, and five months later they were evaluated. Morphological characteristics, productivity, and resource allocation were analyzed with ANCOVA, the nutritional characteristics were analyzed with one-way ANOVA (considering population as a factor). Significant differences (*p* < 0.05) were found for morphological characteristics such as height, number and length of stems, and number of inflorescences. The resource allocation was 13.8% root, 18.4% crown, 29.2% culms + sheaths, 34.8% blades, and 3.8% inflorescence, with no differences between populations (*p* > 0.05). The Puno population stood out for its greater biomass, linked to more stems and inflorescences. Nutritional characteristics varied among populations in terms of crude fiber, neutral detergent fiber, acid detergent fiber, and in vitro dry matter digestibility. These findings are useful for selecting populations in revegetation or genetic breeding programs.

## 1. Introduction

Grasslands constitute one of the major vegetation types worldwide, covering approximately 54% of the Earth’s land surface. They harbor 35% of the planet’s biodiversity and provide essential habitat for 28% of endangered species [1,2]. Dominated by grasses, shrubs, lichens, and, in some cases, scattered trees, grasslands play a crucial role in biodiversity conservation and climate change mitigation [1]. Furthermore, their carbon sequestration capacity makes them a key ally in the fight against global warming, as they can store up to 35% of the Earth’s carbon [2].

In Peru, approximately 22 million hectares are grasslands, representing 57% of the country’s territory. Most are located in the Puna region and are important for generating ecosystem services. Grasslands provide a source of feed for livestock, contribute to water regulation and climate change mitigation, and are also a source of income for local producers [3,4,5]. They are the main food source for ruminants raised in high-Andean conditions, such as alpacas, llamas, and sheep [4,6].

Peruvian high-Andean grasslands exhibit great floristic diversity, composed of different families such as Poaceae, Rosaceae, Asteraceae, Plantaginaceae, Fabaceae, and Cyperaceae [7], of which 46.67% belong to the Poaceae family [3,8]. Within the Poaceae family, one of the most abundant genera is Festuca, with *F. dolichophylla* being one of the most representative species in the high Andean zones [3,9].

*F. dolichophylla* is a high-altitude perennial grass native to the Andes of South America, particularly Peru, Bolivia, Ecuador, and Argentina; it is also found in North and Central America [10]. Its physiological and ecological characteristics allow it to thrive in harsh environments [10], ranging from 3000 to 5000 m above sea level [11]. It grows in different soil types, from strongly acidic to slightly alkaline, with loam, clay loam, and clay textures, influenced by the Andean climate [9,10,12].

Furthermore, *F. dolichophylla* exhibits good biomass accumulation due to its greater height and volume compared to other Festuca species [13]. It has been reported to form mycorrhizal associations, allowing it to store carbon in its stem and bioaccumulate mercury, lead, and arsenic [10]. These characteristics make it a good alternative for revegetation and pasture improvement programs. In other native grasses, such as *Trichloris crinita*, Kozub et al. [14] have demonstrated genetic, morphological, productive, and nutritional variation, making them a viable alternative for use as forage and in the revegetation of degraded areas. *Bouteloua curtipendula* shows high morphological variability among native populations [15], and *F. idahoensis* has demonstrated variability among populations [16], making it a native species with good forage potential. However, to initiate a genetic improvement program and for revegetation purposes, it is important to select specific plants after characterizing several populations under the same conditions.

In different populations of *F. dolichophylla* cultivated under similar conditions, differences in survival rates and growth patterns have been reported [17]. However, few studies exist on the production, resource allocation, morphology, and nutritional characteristics of this species. Therefore, this study was carried out to study the morphology, production, resource allocation, and nutritional content of different populations of *F. dolichophylla* cultivated under similar conditions in the Peruvian Andes. This is important for understanding the behavior of different populations in a common environment; it is also useful for making decisions about their use in revegetation or breeding programs.

## 2. Results

### 2.1. Morphological Characteristics

Plants from Puno showed a greater number of stems and inflorescences than the rest of the populations (*p* < 0.05). For plant height and stem length, Puno plants only surpassed plants from Junín, Pasco, and Huancavelica Community (*p* < 0.05) but showed similar values to plants from Huancavelica University (*p* > 0.05). However, the blade number, blade length, blade width, stem diameter, sheath length, inflorescence length, and spikelet length were similar (*p* > 0.05) between populations, as shown in Table 1.

The mean stem number and inflorescence number of Puno plants were 8.2 and 6.9, respectively, while the stem number was less than or equal to 4.1, and the inflorescence number was less than or equal to 3.1 in the rest of the populations. The plant height and stem length for Puno plants were 441.0 mm and 425.1 mm, respectively; this was statistically similar to plants from Huancavelica University (PH = 340.2 mm, SL = 298.0 mm), as shown in Table 1.

### 2.2. Production and Biomass Allocation

The production of total dry matter, aerial, belowground, blades, culms and sheaths, inflorescence, crowns, and roots were different for each population (*p* < 0.05; Table 2).

The dry matter of aerial, belowground, culms and sheaths, inflorescence, crowns, and roots of Puno plants were higher than in the rest of the populations. The production of blade dry matter form Puno plants showed superiority; however, there were also differences in the rest of the populations; plants from Junín and Huancavelica University showed higher values than those from Huancavelica Community but similar values to those from Pasco.

**Figure 1:**
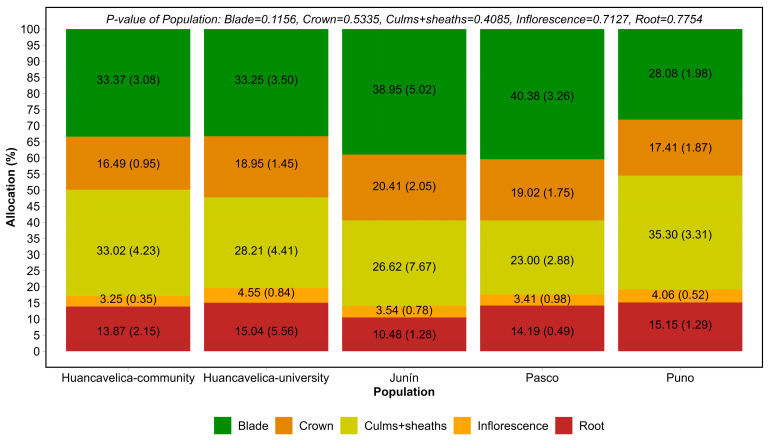
Dry matter allocation (standard error) to different organs in different *F. dolichophylla* populations grown under similar conditions (*n* = 5 for population).

Despite differences in dry matter production in the different organs, when expressed as a percentage of allocation to the different plant organs (dry matter allocation), no differences (*p* > 0.05) were found between populations for the allocation to blades, culms and sheaths, inflorescence, crowns, and roots (Figure 1).

The mean (standard error) of dry matter allocation of *F. dolichophylla* was 13.75 (1.19), 18.45 (0.73), 29.23 (2.16), 34.81 (1.69), and 3.76 (0.31) % to root, crown, culms and sheaths, blades, and inflorescence, respectively.

### 2.3. Nutritional Composition

Crude fiber, neutral detergent fiber, acid detergent fiber, and in vitro dry matter digestibility were influenced by population (*p* < 0.05). According to the Tukey test, the crude fiber content in plants from Puno exceeded that in the plants from Junín and Pasco but was similar to those from Huancavelica Community and University. The neutral detergent fiber of *F. dolichophylla* from Puno was superior only to those from Pasco, while the acid detergent fiber of plants from Puno was superior only to those from Junín. Although an influence of populations for in vitro dry matter digestibility was found (*p* < 0.05), no differences between populations were found according to the Tukey test (Table 3). On the other hand, the dry matter percentage, ash, ether extract, crude protein, nitrogen-free extract, and gross energy did not show differences between populations (*p* > 0.05).

## 3. Discussion

*F. dolichophylla* differed among populations in plant height, number of stems, stem length, and number of inflorescences. Plant height in *T. crinita* [18], *F. idahoensis* [16], *B. curtipendula* [15], and *Chloris gayana* [19] also showed intraspecific variability, which is consistent with our results. Gil et al. [18] reported intraspecific differences for the number of tillers of *T. crinita*, while May et al. [16] found variation in the number of flower stems of *F. idahoensis*. The number of inflorescences has been different between populations of *T. crinita* [18] and different ecotypes of *B. curtipendula* [15], indicating that these characteristics are dependent on the species and population [20].

Plant growth is determined by the rate of photosynthetic carbon uptake, carbon loss, and the accumulation of photo-assimilates in different plant compartments, which gives rise to specific morphologies for each population [21]; and this is determined by the genetic constitution of each population and the environment in which it has developed [11]. Puno plants showed superiority, with approximately more than 1.5 times the number of leaves compared to the rest of the populations; although no significant differences were found for this trait (Table 1), this would give them an advantage in biomass accumulation by photosynthesis.

The area of one leaf, considering the blade as a triangle (BW × BL/2), would be 121.62, 114.78, 134.62, 85.14, and 140.40 mm^2^ for plants from Huancavelica Community, Huancavelica University, Junín, Pasco, and Puno, respectively, with Puno highlighted as having the highest value. The number of leaves on plants from Puno was 1.58 times greater than those from Pasco, while the leaf area was 1.65 times greater. The number of leaves on plants from Puno was 2.12 times greater than those from Huancavelica Community, while the leaf area was only 1.15 times greater. The higher leaf area of plants from Puno would give them a greater advantage in photosynthesis compared to the rest of the populations.

Plants from Puno have a greater number of inflorescences, which is consistent with their greater number of stems (Table 1). Gonzales et al. [20] reported that native grasses reproduce preferentially by seeds and introduced species have a greater reproduction by tillering. Puno plants would have reproduction through seeds as an important complementary strategy along with vegetative reproduction. Castro et al. [22] reported a germination rate of 18% for *F. dolichophylla* seeds, a considerable value for a native grass, supporting the importance of seed reproduction for this species. The results of this study did not show differences in the length of the inflorescence for *F. dolichophyla*. In other grasses, such as *Agrostis capillaris* [23] and *B. curtipendula* [15], differences in inflorescence length were evident between accessions and ecotypes, respectively.

The total biomass production of the plant and its parts (roots, crowns, inflorescence, culms and sheaths, and blades) was higher in Puno plants. Flores [24] reported aerial dry matter yields between 80 and 190 g for small plants of *F. dolichophylla* from two different sites in Puno; however, the production of aerial dry matter for plants from Puno in this experiment was 17.83 g, which is lower than the data for Flores [24]. Our results come from plants that had twelve months of establishment and five months of growth; however, the results for Flores [24] come from native plants, which would have a longer growth period. Similarly, in Junín, Yaranga et al. [12] reported yields for individual *F. dolichophylla* plants of 116 ± 19.4 g under natural conditions. Additionally, the superior biomass production of the Puno plants was due to the different environmental conditions of the plants’ origin sites; the Puno plants came from an environment with 6.9 °C, 677.0 mm of precipitation, and the lowest nitrogen and phosphorus content; these different characteristics would contribute to the differentiation between populations, as demonstrated for *F. idahoensis* [16].

Variability in aboveground biomass production has been reported among populations of *T. crinita* [18,25], between accessions of *C. gayana* [19], and between ecotypes of *B. curtipendula* [15], which agrees with our results for variability between populations of *F. dolichophylla*, but in this case, in a common environment and with uniform management. In *F. idahoensis*, variation was reported between populations for seed production [16]; in our case, we also found differences between populations of *F. dolichophylla* for inflorescence weight.

The resource allocation to the different organs of the plant in *F. dolichophylla* did not show differences between populations. More than 65% of the resources were allocated to the aerial part, and the remainder was assigned to the crown and root in all populations (Figure 1). In *F. dolichophylla* from Puno, 5% was assigned to roots, 86% to stems, and 9% to leaves [24]; this differs from our results. While our results come from young plants (one year of establishment and five months of growth), Flores [24] studied plants with natural growth, which would be older than ours; furthermore, Flores [24] did not report assignment to the crown and inflorescence.

Typically, under adverse weather conditions, such as cold temperatures and seasonal droughts, perennial plants often invest more in the belowground part than in the aboveground part [21]; in our study, this behavior was not evident, since the plants were young and small (total biomass between 4.47 and 26.19 g—Table 2) and this could change as the plants mature and become larger; also, our results had different variability levels (standard error, Figure 1) for dry matter allocation. In *F. orthophylla*, Monteiro et al. [21] reported an accumulation of 46% to the aerial biomass in larger plants (biomass greater than 400 g).

In *T. crinita*, no differences were evident in the resource allocation to the aerial part under different levels of shade [25]; resource allocation to the panicle, leaf, and stems was similar between populations of *Tridens flavus*, but allocation to the seeds was different [26]. Instead, results showed greater resource allocation to the roots for exotic grasses and greater allocation to the crown in native species [27]; other studies showed greater allocation of biomass to roots in native species, compared to introduced species [28]. For *F. dolichophylla*, no differences are shown for resources allocation, despite the fact that plants from Puno, Junín, Pasco, and Huancavelica University were introduced into the environment of Huancavelica Community, indicating that this characteristic is specific to each species.

The results for the dry matter percentage, minerals, neutral detergent fiber, and acid detergent fiber in this study were lower than those reported by Mamani-Linares and Cayo-Rojas [29] for *F. dolichophylla*, but our results for crude protein were higher. The reports of Mamani-Linares and Cayo-Rojas [29] come from a study conducted in Bolivia; their results in April for crude protein, dry matter percentage, ash, neutral detergent fiber, and acid detergent fiber were 5.46%, 54.22%, 5%, 73.82%, and 51.11%, respectively. On the other hand, Mamani et al. [30] reported a crude protein content of 5.1% and neutral detergent fiber of 63.94% for *F. dolichophylla* from Puno; these results come from an experiment without adding organic matter, as we did. The differences would be mainly due to the different study environments, age of the plants and study seasons (dry, rainy), and the objectives pursued in each of them; Mamani–Linares and Cayo-Rojas [29] evaluated the effect of the season, while Mamani et al. [30] studied the effect of revegetation with the addition of organic matter. Merlo-Maydana et al. [31], in a study on *F. dolichophylla* in Bolivia, reported that the crude protein was higher in the rainy season, compared to the dry season; in addition, the neutral detergent fiber, acid detergent fiber, and lignin content increase with age, while the crude protein decreases with the age of the plant.

Differences in nutritional characteristics between ecotypes and populations have been reported in other species. In *Pennisetum ciliare*, cultivated in the same plot, variability was evident between the ecotypes [32], while in *T. crinita*, differences between populations were reported [18] in the same environment, which agree with our results in *F. dolichophylla*. On the other hand, all the evaluated characteristics showed different degrees of variability of characteristics, evidenced in their standard error.

The variation found in the morphological, productive, biomass allocation, and nutritional characteristics would be mainly due to the variations in the genetic constitution between populations of *F. dolichophylla*, since the environment and management were similar; this is consistent with reports by May et al. [16] in *F. idahoensis*, who reported variation within and between populations for morphological characteristics in a study in a uniform environment. This indicates that the specific adaptation of plants to their native environment can determine differences in their agromorphological characteristics, affecting their growth, defense, and future resilience [33]. For productive characteristics, *F. dolichophylla* from Puno showed superiority to the rest of the populations; this is in agreement with Esteban et al. [34], and May et al. [16], who demonstrated variation between populations for dry matter production of *T. crinita* and for seed weight of *F. idahoensis*, respectively.

The adaptation of each population to its environment of origin would cause a differentiated expression in various characteristics of the plants [33]; Bertiller et al. [35] confirmed that soil characteristics were associated with *F. pallescens* coverage in Argentina. In the present study, the environmental variables (soil, precipitation, and temperature) showed differences between the places of origin of each population [17], which would have shaped the genetic differentiation of each population. Variation in morphological and genetic characteristics is very important for developing breeding and conservation programs for native grasses; also, these differences are key in their sustainable management in livestock systems and conservation of the Andean ecosystem [11,36].

Understanding the intraspecific variation is very important for the proper management of *F. dolichophylla*, considering that morphological diversity is the result of the complex interaction between genetic and environmental factors [37]. The results of this research demonstrate differences between populations in production and some morphological and nutritional characteristics in *F. dolichophylla* grown under similar conditions and management, but there were no differences for resource allocation. However, further studies in different environments and genetic constitution are necessary for better decision-making regarding management, conservation, and eventual genetic breeding.

## 4. Materials and Methods

### 4.1. Populations and Experimental Plot

Five populations of *F. dolichophylla* were studied: Pastales Huando Peasant Community, Huancavelica (Huancavelica Community), Lachocc South American Camelid Research and Development Center of the National University of Huancavelica (Huancavelica University), Junín, Pasco, and Puno. Temperature averages were 8.1, 7.8, 9.1, 9.9, and 6.9 °C [38], and the annual rainfall was 899.0, 911.0, 573.9, 1020.4, and 677.0 mm for Huancavelica Community, Huancavelica University, Junín, Pasco, and Puno, respectively [39]. The altitude ranges of the collected populations varied from 3930 to 4666 m.a.s.l., and variation in soil characteristics was also shown. The means of nitrogen content was 0.27, 0.32, 0.22, 0.55, and 0.06%; the phosphorus content was 20.03, 12.36, 7.87, 11.06, and 8.36 ppm; the potassium content was 257.60, 275.80, 168.80, 166.80, and 985.27 ppm; and the pH was 5.51, 4.77, 6.81, 4.57, and 6.83 for Huancavelica Community, Huancavelica University, Junín, Pasco, and Puno, respectively. The lowest nitrogen and phosphorus contents were found in Puno and Junín; however, the lowest potassium contents were found in Junín and Pasco, while the pH ranged from 4.57 to 6.83. These data were reported as ranges by Paucar et al. [17].

**Figure 2:**
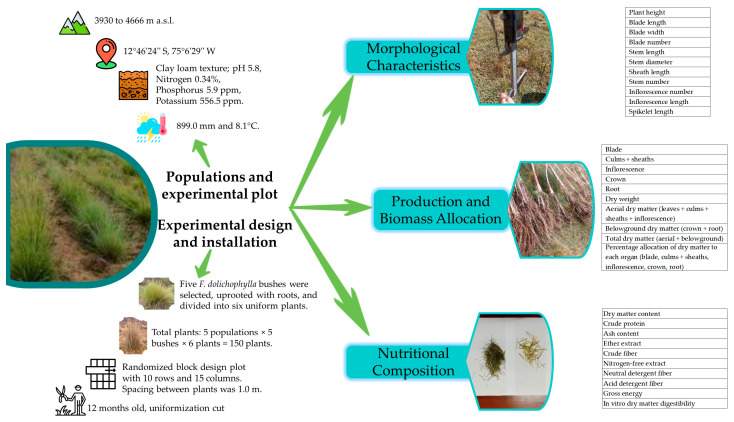
Study methodology.

The experimental plot was located in Huancavelica Community (12°46′24″ south latitude and 75°6′29″ west longitude), at an altitude of 4620 m.a.s.l. The annual precipitation and average temperature were 899.0 mm [39] and 8.1 °C [38], respectively. The greatest rainfall occurs between October and April, coinciding with the highest average temperatures; while from May to September, rainfall is lowest and coincides with the lowest temperatures. The soil of the experimental plot was uniform, with a clay loam texture; the average pH, nitrogen, phosphorus, and potassium contents were 5.8, 0.34%, 5.9 ppm, and 556.5 ppm, respectively (Figure 2).

### 4.2. Experimental Design and Installation

From each population, five *F. dolichophylla* bushes were selected based on their good vigor. They were extracted with all their roots and divided into six small plants of uniform size. Considering five populations, five bushes per population, and six plants per bush, in total, 150 plants (5 × 5 × 6 = 150) were initially installed. These plants were installed in an experimental plot (10 rows and 15 columns), with a spacing of 1.0 m between plants, under a randomized block design; more details can be found in Paucar et al. [17]. After 12 months of establishment, the uniformization cut was made, and the initial number of leaves was recorded (Figure 2).

### 4.3. Morphological Characteristics

After the establishment period, there were different mortality rates for different populations [17]; after the uniformization cut, the plants were allowed to grow for a period of five months (January–May). After establishment and the growth period, 85 plants survived, so measurements were taken based on these plants. The plant height (PH) was considered as the height to the flag leaf, then the length (BL) and width (BW) of its blade was measured. The BL was measured from the ligule to the apex, and the BW was measured in the expanded blade, next to the ligule; in addition, the number of blades (BN) on each plant was recorded. The stem length (SL) was considered from the ground to the inflorescence node, the stem diameter (SD) was measured above the first node, the sheath length (ShL) was considered from the node to the ligule, and the stem number (SN) was also counted. All visible inflorescences were counted (IN), and the length of the inflorescence (IL) and spikelet (SpL) on the tallest stem was measured. Length measurements were performed with a digital Vernier (Figure 2).

### 4.4. Production and Biomass Allocation

Five plants were randomly selected from each population. The aerial parts of these plants were carefully wrapped with raffia to avoid losses during transport and were extracted with all their roots. The soil from the root was removed by washing in running water and transferred to the Grassland Laboratory of the National University of Huancavelica for further processing.

Each plant was separated into its blade, culms and sheaths, inflorescence, crown, and roots. The blades were cut from the ligule; the culms and sheaths were separated from the inflorescence node to 2.0 cm above the place where the root began. The roots were separated from the remainder, and the rest was considered the crown. All samples were dried at 60 °C for 48 h in an oven, after which the dry weights were obtained on an analytical balance with a readability of 0.1 mg. Dry matter production was expressed in grams; aerial (leaves, culms, sheaths, and inflorescence) and belowground (crown and root) dry matter was also considered.

The allocation of dry matter to the different organs (the blade, culms and sheaths, inflorescence, crown, and root) was considered as the percentages that these organs represented of the total weight (Figure 2).

### 4.5. Nutritional Composition

The nutritional content was determined from the aerial part of the plants. For this purpose, all the plants were harvested, and five groups were formed for each population, taking into account that each group contained plants from the same original plant (Figure 2). This was necessary to complete the sufficient amount of dry matter for subsequent analyses.

To determine the dry matter content (DMP), the gravimetry method was used [40]; crude protein (CP) was calculated using the 928.08 method (Kjeldahl digestion). The gravimetric method was used to determine the ash content, extract ether—EE was determined by ether extraction method [41]. The crude fiber (CF) content was determined using the Ankom system [42], and the nitrogen-free extract (NFE) was calculated according to AOAC [41]. The neutral detergent fiber (NDF) and acid detergent fiber (ADF) content were determined following the methodology of Van Soest et al. [43]. The gross energy (GE) content was determined from the energy contributions of crude protein, crude fiber, ether extract, and nitrogen-free extract [44]. Finally, the in vitro dry matter digestibility (IVDMD) was determined using the DAYSI II equipment [45].

### 4.6. Data Analysis

An exploratory data analysis was performed to verify the outliers, the assumptions of normality were verified with the Shapiro–Wilk test and homoscedasticity with the Levene test, and the results showed compliance with both. Therefore, an analysis of covariance of the morphological characteristics was performed, including populations, the nested effect of bush within population and blocks (rows) as factors, and the initial number of leaves was taken into account as a covariate. Productive characteristics and resource allocation were analyzed using a linear model that included populations as a factor and the initial number of leaves as a covariate. Nutritional characteristics were analyzed using a one-way design, including populations as the only factor. When differences were found between populations during the analysis, the comparison of means was performed using the Tukey method. In all cases, a significance level of 0.05 was used and R. v. 4.4.0 software was used [46] for the analysis.

## 5. Conclusions

Some morphological characteristics (plant height, stem number and length, inflorescence number), all productive characteristics (biomass of different plant organs), and some nutritional characteristics (crude fiber, neutral detergent fiber, acid detergent fiber, and in vitro dry matter digestibility) of *F. dolichophylla* showed variability between populations; however, the percentage of resource allocation to the different organs of the plant was similar. All variables studied showed different ranges of variation (standard errors); this variation and differences found would be mainly due to the different genetic constitution of each population, since the management and environment were similar. These findings highlight the behavior of different populations of *F. dolichophylla* cultivated in common environment, which is important for making decisions for use in revegetation or breeding programs.

## Figures and Tables

**Table 1:** Means ± SE (standard error) of morphological characteristics of different populations of *F. dolichophylla* grown under similar conditions.

Parameter	*p*-Value *	Huancavelica Community	Huancavelica University	Junín	Pasco	Puno	Total
Blade							
PH (mm)	0.0019	261.93 ± 29.10 b	340.15 ± 33.80 ab	217.05 ± 11.63 b	199.67 ± 20.15 b	441.02 ± 15.42 a	296.53 ± 13.80
BN (n)	0.0924	35.24 ± 6.53 a	38.42 ± 7.79 a	43.44 ± 11.96 a	47.42 ± 8.63 a	74.80 ± 7.66 a	49.44 ± 4.08
BL (mm)	0.2360	86.56 ± 8.76 a	96.05 ± 9.73 a	108.13 ± 8.86 a	73.08 ± 7.14 a	93.29 ± 6.09 a	90.74 ± 3.72
BW (mm)	0.1810	2.81 ± 0.17 a	2.39 ± 0.08 a	2.49 ± 0.12 a	2.33 ± 0.14 a	3.01 ± 0.11 a	2.66 ± 0.07
Culm							
SN (n)	0.0048	4.05 ± 0.69 b	3.64 ± 1.00 b	2.93 ± 0.38 b	3.08 ± 0.65 b	8.16 ± 0.86 a	4.87 ± 0.42
SL (mm)	0.0116	241.17 ± 26.72 b	298.01 ± 37.68 ab	247.61 ± 36.45 b	222.81 ± 26.14 b	425.05 ± 20.33 a	300.86 ± 15.27
SD (mm)	0.2320	2.00 ± 0.11 a	2.00 ± 0.13 a	1.79 ± 0.10 a	1.63 ± 0.12 a	2.19 ± 0.10 a	1.96 ± 0.05
ShL (mm)	0.0509	130.88 ± 9.82 a	154.75 ± 19.19 a	116.14 ± 10.01 a	98.70 ± 8.95 a	154.37 ± 6.13 a	133.53 ± 4.94
Inflorescence							
IN (n)	0.0048	3.09 ± 0.38 b	3.00 ± 0.83 b	2.43 ± 0.33 b	2.92 ± 0.65 b	6.88 ± 0.77 a	4.06 ± 0.35
IL (mm)	0.3130	96.69 ± 5.47 a	113.9 ± 11.23 a	91.82 ± 6.50 a	80.83 ± 11.23 a	108.20 ± 4.95 a	99.08 ± 3.34
SpL (m)	0.2830	8.42 ± 0.26 a	9.55 ± 0.46 a	8.27 ± 0.30 a	7.69 ± 0.21 a	9.14 ± 0.38 a	8.64 ± 0.17
N		22	11	14	13	25	85

*, *p*-value to evaluate the effect of the populations; ab in each row show significant differences according to the Tukey test (α = 0.05). PH, plant height; BN, blade number; BL, blade length; BW, blade width; SN, stem number; SL, stem length; SD, stem diameter; ShL, sheath length; IN, inflorescence number; IL, inflorescence length; SpL, spikelet length.

**Table 2:** Means ± SE (standard error) of dry biomass from different organs in grams of different populations of *F. dolichophylla*, grown under similar conditions (*n* = 5 per population).

	Aerial	Belowground	Blade	Culms and Sheaths	Inflorescence	Crown	Root
*p*-value *	<0.001	<0.001	<0.001	<0.001	0.001	<0.001	<0.001
Huancavelica Community	3.20 ± 0.99 b	1.27 ± 0.31 b	1.47 ± 0.46 c	1.60 ± 0.60 b	0.14 ± 0.04 b	0.71 ± 0.20 b	0.55 ± 0.13 b
Huancavelica University	7.98 ± 4.26 b	3.40 ± 1.96 b	4.18 ± 2.52 b	3.37 ± 1.67 b	0.44 ± 0.20 b	1.84 ± 0.89 b	1.55 ± 1.10 b
Junín	6.39 ± 2.10 b	3.13 ± 1.24 b	4.19 ± 1.84 b	1.92 ± 0.44 b	0.28 ± 0.07 b	2.09 ± 0.83 b	1.04 ± 0.41 b
Pasco	4.47 ± 1.35 b	2.37 ± 0.80 b	2.62 ± 0.80 bc	1.55 ± 0.44 b	0.30 ± 0.15 b	1.42 ± 0.51 b	0.95 ± 0.29 b
Puno	17.83 ± 3.67 a	8.36 ± 1.45 a	7.37 ± 1.46 a	9.40 ± 2.28 a	1.06 ± 0.27 a	4.56 ± 0.98 a	3.79 ± 0.60 a
Total	7.98 ± 1.55	3.71 ± 0.72	3.97 ± 0.77	3.57 ± 0.81	0.44 ± 0.09	2.13 ± 0.40	1.58 ± 0.34

*, *p*-value to evaluate the effect of the populations; abc in each column indicate significant differences according to the Tukey test (α = 0.05).

**Table 3:** Means ± SE (standard error) of nutritional characteristics of different populations of *F. dolichophylla* grown under similar conditions (*n* = 5 for population).

Parameter	*p*-Value *	Huancavelica Community	Huancavelica University	Junín	Pasco	Puno	Total
DMP (%)	0.9830	36.22 ± 1.43 a	34.65 ± 1.48 a	36.24 ± 3.05 a	36.18 ± 3.00 a	36.12 ± 1.67 a	35.88 ± 0.93
Ash (%)	0.3390	3.94 ± 0.56 a	3.49 ± 0.57 a	4.75 ± 0.31 a	3.79 ± 0.17 a	4.24 ± 0.43 a	4.04 ± 0.20
EE (%)	0.6480	2.16 ± 0.28 a	1.58 ± 0.45 a	1.85 ± 0.29 a	2.27 ± 0.40 a	1.8 ± 0.30 a	1.93 ± 0.15
CP (%)	0.8990	7.44 ± 0.67 a	8.05 ± 1.20 a	7.44 ± 0.87 a	7.19 ± 0.80 a	8.31 ± 1.00 a	7.69 ± 0.39
CF (%)	0.0038	37.79 ± 1.16 ab	37.94 ± 0.62 ab	35.66 ± 0.34 b	35.04 ± 0.71 b	39.19 ± 0.60 a	37.12 ± 0.43
NDF (%)	0.0035	66.57 ± 0.51 ab	70.1 ± 0.87 a	68.47 ± 1.21 ab	65.1 ± 1.01 b	69.24 ± 0.44 a	67.89 ± 0.51
ADF (%)	0.0149	42.83 ± 1.26 ab	42.68 ± 0.78 ab	39.15 ± 1 b	39.45 ± 1.40 ab	43.57 ± 0.39 a	41.54 ± 0.57
NFE (%)	0.0886	48.68 ± 1.83 a	48.93 ± 1.69 a	50.29 ± 0.70 a	51.71 ± 0.97 a	46.46 ± 0.75 a	49.22 ± 0.63
IVDMD (%)	0.0331	39.71 ± 1.39 a	38.8 ± 1.23 a	42.64 ± 1.23 a	42.77 ± 0.71 a	39.02 ± 0.67 a	40.59 ± 0.57
GE (Kcal/100 g)	0.5440	425.12 ± 2.67 a	424.85 ± 2.30 a	420.07 ± 3.28 a	425.90 ± 2.92 a	423.23 ± 1.49 a	423.84 ± 1.14

*, *p*-value to evaluate the effect of the populations; ab in each row indicate significant differences according to the Tukey test (α = 0.05). DMP, dry matter percentage; EE, ether extract; CP, crude protein; CF, crude fiber; NDF, neutral detergent fiber; ADF, acid detergent fiber; NFE, nitrogen-free extract; IVDMD, in vitro dry matter digestibility; GE, gross energy.

## Data Availability

The data presented in this study are available on request from the corresponding author.
